# Chronic constrictive pericarditis: a rare cardiac involvement in primary Sjögren’s syndrome

**DOI:** 10.1186/s12872-023-03491-6

**Published:** 2023-09-20

**Authors:** Fabiana Duarte, Luís Oliveira, Tomás Fontes, Sância Ramos, Raquel Dourado, Dinis Martins

**Affiliations:** 1 Cardiology Department, Hospital of Divino Espírito Santo of Ponta Delgada, EPER, São Miguel Island, Avenida D. Manuel I 9500-370, Azores, Portugal; 2 Rheumatology Department, Hospital of Divino Espírito Santo of Ponta Delgada, EPER, São Miguel Island, Avenida D. Manuel I 9500-370, Azores, Portugal; 3 Anatomical Pathology Department, Hospital of Santa Cruz, Centro Hospitalar, Lisboa Ocidental, Portugal

**Keywords:** Constrictive pericarditis, Autoimmune disease, Primary Sjögren’s syndrome, Echocardiography, Calcification, Partial pericardiectomy, Case report

## Abstract

**Background:**

Constrictive pericarditis represents a chronic condition and systemic inflammatory diseases are a known, yet uncommon, cause. Pericardial involvement is seldom reported in primary Sjögren’s syndrome, usually occurring in association with pericardial effusion or pericarditis.

We report a case of constrictive pericarditis with an insidious course and unusual evolution associated with primary Sjögren’s syndrome. Due to the challenging nature of the diagnosis, clinical suspicion and multimodality imaging are essential for early identification and prompt initiation of treatment. Long-term outcomes remain uncertain.

To the best of our knowledge, no other cases linking this autoimmune disease to constrictive pericarditis have been reported.

**Case presentation:**

We present the case of a 48-year-old male patient with moderate alcohol habits and a history of two prior hospitalizations. On the first, the patient was diagnosed with primary Sjögren’s syndrome after presenting with pleural effusion and ascites, and empirical corticosteroid regiment was initiated. On the second, two-years later, he was readmitted with complaints of dyspnea and abdominal distension. Thoracic computed tomography revealed a localized pericardial thickening and a thin pericardial effusion, both of which were attributed to his rheumatic disease. A liver biopsy showed hepatic peliosis, which was considered to be a consequence of glucocorticoid therapy. Diuretic therapy was adjusted to symptom-relief, and a tapering corticosteroid regimen was adopted.

Four years after the initial diagnosis, the patient was admitted again with recurrent dyspnea, orthopnea and ascites. At this time, constrictive pericarditis was diagnosed and a partial pericardiectomy was performed.

Although not completely asymptomatic, the patient reported clinical improvement since the surgery, but still with a need for baseline diuretic therapy.

**Conclusion:**

Albeit uncommon, connective tissue disorders, such as primary Sjögren’s syndrome, should be considered as a potential cause of constrictive pericarditis, especially in young patients with no other classical risk factors for constriction.

In this case, after excluding possible infectious, neoplastic and autoimmune conditions, a primary Sjögren´s syndrome in association with constrictive pericarditis was assumed. This case presents an interesting and challenging clinical scenario, highlighting the importance of clinical awareness and the use of multimodal cardiac imaging for early recognition and treatment.

**Supplementary Information:**

The online version contains supplementary material available at 10.1186/s12872-023-03491-6.

## Background

Constrictive pericarditis (CP) is a chronic condition consisting of pericardial fibrous thickening caused by persistent inflammation, resulting in impaired diastolic ventricular filling. Tuberculosis is the leading cause globally, although in developed countries it tends to be more idiopathic or a result of viral infections. CP can also occur as a consequence of systemic inflammatory diseases, which account for 3–7% of cases, and it requires a high level of clinical awareness. In some cases, a specific etiology cannot be identified [1–4].

Pericarditis (acute and recurrent) is the most common form of cardiac involvement in rheumatic connective tissue diseases (CTD) [5, 6]. In fact, systemic lupus erythematosus (SLE) courses with asymptomatic pericardial effusion in more than 50% of patients, with 25% developing symptomatic pericarditis [5, 7, 8]. In rheumatoid arthritis (RA), the prevalence of asymptomatic effusion ranges from 30 to 50%, whereas acute pericarditis occurs in less than 10% of cases. In systemic sclerosis (SSc), the prevalence of pericarditis ranges from 6 to 16% [5, 9–12]. Pericardial involvement is commonly observed in systemic vasculitis, particularly in medium and ANCA-associated small vessel vasculitis, such as eosinophilic granulomatosis with polyangiitis (20–25%) and granulomatosis with polyangiitis (< 10%) [5, 13–15]. It is rarely associated with large vessel vasculitis, as Takayasu´s arteritis (< 10%) [5, 6, 16]. Pericardial involvement can also occur in autoinflammatory syndromes, sarcoidosis and IgG4-related disease [5, 6, 17, 18]. In Sjögren’s syndrome, cardiac involvement is rare and usually asymptomatic [19]. Constrictive pericarditis is a rare finding in these conditions and can be a complication of indolent and subclinical pericardial involvement [5, 6, 20–22].

Clinical manifestations of CP are quite variable, ranging from a silent presentation to overt heart failure symptoms, making diagnosis troublesome at times. Clinical suspicion should arise in the presence of peripheral edema, hepatomegaly, pleural effusion and typically Kussmaul sign and pericardial knock [1, 2]. In workup diagnosis, electrocardiogram (EKG) and laboratory findings are not specific, and imaging methods are required to confirm the diagnosis. Transthoracic echocardiography (TTE) should be the initial imaging method and it has a high diagnostic yield for CP with several specific findings. Computed tomography (CT) and cardiac magnetic resonance can help in providing supportive diagnostic features and in differential diagnosis [2, 3].

In chronic CP, medical therapy is used for symptoms palliation, while surgical pericardiectomy is the only definitive treatment option. Early surgical intervention is essential for clinical improvement and for a favorable long-term outcome [1, 2, 4].

## Case presentation

We report the case of a 48-year-old male patient with a past medical history of obesity and moderate alcohol consumption, but no history of smoking or other illicit drugs habits. His father died at the age of 63 due to end-stage liver disease. No other relevant family disorders or cardiovascular diseases were reported.

He was first admitted to the ward at the age of 41 years old due to a diffuse serosal effusion with a pleural and ascitic predominance. He reported a history of progressively worsening abdominal distention and shortness of breath for the two months prior. The patient did not report any previous infections, travel abroad or lifestyle changes. No other cardiovascular symptoms, such as chest pain or loss of consciousness were referred. A history of sicca symptoms was cleared out, including dry eyes that required eye drop moisturizer, and also dry skin. No other symptoms were reported, including skin changes, mucosal ulceration, hair loss, Raynaud’s phenomenon, salivary gland swelling, joint tenderness or swelling, proximal muscle weakness, neurological or constitutional symptoms, or any pattern of fever. At examination, the patient presented with diffuse edema, most prominently at the abdomen, and diminished breath sounds with dullness to chest percussion. No other relevant changes were evident.

The combination of the presented clinical manifestations and the young age of onset prompted an in-depth etiological investigation. The admission EKG showed sinus rhythm with no acute or dynamic changes. The initial workup diagnosis included a wide range of blood tests, including serological and immunological markers. The blood count, metabolic panel and other biochemical markers showed no significant alterations, except for a C-reactive protein level of 6 mg/dL (normal < 0.3 mg/dL) and mild liver cytolysis. The serum protein (4.6 g/dL) and albumin (2.3 g/dL) levels were both low. The 24-h urinalysis was also normal. Immunofixation was negative, and serum IgG, IgA, IgM, light chains kappa and lambda and protein electrophoresis were normal. Further serological and molecular testing looking for an infectious etiology yielded negative results, having been searched the following microorganisms: *Bartonella* spp., *Coxiella* burnetii, *Brucella* spp., *Borrelia* spp, *Chlamydia* spp. and *Mycobacterium tuberculosis* spp. Specific immunological tests were performed. Antinuclear antibody immunofluorescence assay was positive at a titer of 1:320. The anti-ENA ELISA immunoassay revealed a positive anti-SS-A/Ro at a titer of 98.0 U/mL (reference < 7 U/mL), but no other specific antibody positivity was found (including the mixed connective tissue disease hallmark anti-U1-RNP). The anti-cyclic citrullinated peptides antibodies, rheumatoid factor, anti-dsDNA and ANCA antibodies were negative. Complement fractions C3 and C4 were not consumed (Table 1).

**Table 1:** Timeline table for the main laboratory findings

*First hospital admission*		
***Laboratory analysis***	**Results**	**Normal range / Result**
*Hemoglobin (g/dL)*	12.6	14.0 – 18.0
*Hematocrit (%)*	37.7	40 – 52
*Mean corpuscular volume (fL)*	86.6	80 – 96
*Platelets (*× *10*^*3*^*/µL)*	348	150 – 430
*Leukocytes (*× *10*^*3*^*/µL)*	9.79	4.0 – 11.5
*aPTT, PT (sec)*	Normal	–
*Lupus anticoagulant (ratio)*	1.17	0.8 – 1.2
*Renal function*	Normal	–
*Metabolic panel*	Normal	–
*AST/ ALT/ GGT (U/L Total bilirubin (mg/dL)*	49 / 117 / 133 1.16	< 37, < 78, 46 – 116, 15 – 85 0.2 – 1.0
*Thyroid function*	Normal	–
*C-reactive protein (mg/dL)*	**6**	< 0.3
*Serum total proteins (g/dL)*	4.6	6.0 – 8.2
*Serum albumin (g/dL)*	2.3	3.4 – 5
*24-h urinalysis*	Normal	–
***Tumor markers***		
* CA19.9, β2M, βhCG, AFP, PSA, NSE, CEA*	Negative	–
***Immunological tests***		
*** ANA IF assay***	**1:320**	Positive
*** Anti-ENA screening (U/mL)***	**Anti-SS-A/Ro positive** (98.0) Anti-SM, anti-SS-B(La), anti-RNPU1, anti-Scl70, anti-CENP B, anti-Jo1 (negative)	< 7 U/mL (anti-SS-A)
* Anti-dsDNA (IU/mL)*	10.00	Negative
* ANCA-MPO (IU/mL)*	< 0.20	Negative
* ANCA-PR3 (IU/mL)*	< 0.20	Negative
* Anti-U1-RNP (U/mL)*	0.80	Negative
* Anti-CCP (U/mL)*	< 0.40	Negative
* Rheumatoid factor (UI/mL)*	< 11	0 – 15
***Serum immunoglobulins***		
* IgA (mg/dL)*	256	70 – 400
* IgM (mg/dL)*	90	40 – 230
* IgG (mg/dL)*	1250	700 – 1600
* Light chains kappa (mg/dL)*	305	170 – 370
* Light chains lambda (mg/dL)*	183	90 – 210
* Ratio light chains K/L*	1.67	Normal
* Protein electrophoresis*	Normal	–
* C3 / C4 fractions (mg/dL)*	167 / 33	90 – 180 / 10 – 40
***Serological assays***		
* Rapid Plasma Reagin*	Negative	–
* HIV, HCV, HBV, CMV, EBV*	Negative	–
* Toxoplasmosis*	Negative	–
* Anti-Treponema pallidum*	Negative	–
* Bacterial infections panel*^*a*^	Negative	–
***Pleural effusion analysis***^*b*^		
* Criteria of transudate. No malignant cells. Negative cultures*		
*** Tissue biopsy***		
* Pleural biopsy*	Nonspecific inflammatory changes	
* Minor salivary gland biopsy*	Small infiltrate of lymphocytes (periductal)	
***Second hospital admission***		
* Pleural fluid analysis*^*b*^	Transudative effusion	
* Liver biopsy*	Hepatic peliosis, sinusoidal congestion, mild ductular reaction (histopathological findings)	

^a^*Bartonella* spp, *Coxiella* burnetii, *Brucella* spp, *Borrelia* spp, *Chlamydia* spp. and *Mycobacterium tuberculosis* spp

^b^Biochemical, cytological and microbiological analysis

The imaging workup included a chest X-ray that revealed bilateral pleural effusion. An abdominal computed tomography for additional clarification showed hepatomegaly with diffuse heterogeneity, splenomegaly and a diffuse subcutaneous cellular edema. A TTE was performed, but no significant pathological findings were reported.

The analysis of pleural and ascitic fluid revealed a transudative effusion, and cytological analysis excluded the presence of malignant cells. A pleural biopsy revealed nonspecific reactive inflammatory changes, with no evidence of pleural thickness or fibrosis. A Schirmer test of 3 mm in 5 min bilaterally proved an abnormal ocular dryness for this particular patient, in the absence of secondary causes for it. Minor salivary gland biopsy showed no signs of lymphocytic sialadenitis.

During this first hospitalization, a diagnosis of primary Sjögren’s syndrome (pSS) was made, and 2016 ACR/EULAR classification criteria for pSS were also fulfilled with a total score of 4 and no exclusion criteria present [10]. The patient was started on prednisolone 60 mg/day and was discharged after 30 days of hospitalization with no symptoms.

The patient remained clinically stable for two years, during which he did not fully adhere to follow-up appointments, hindering treatment adjustments and optimization. After that time, the patient was readmitted due to dyspnea and abdominal distension. Hepatosplenomegaly and pleural effusion were detected, and a liver biopsy revealed hepatic peliosis, which was interpreted as a consequence of glucocorticoid therapy. A new thoracic CT scan was performed, revealing a localized pericardial thickening and a thin circumferential pericardial effusion, but a new TTE was not performed at that time. With no infectious or neoplastic cause underneath and no other symptoms suggesting a major involvement of a systemic rheumatic disease, diuretic therapy was adjusted to symptom-relief. The patient continued to receive medical surveillance through dedicated consultations and gradually reduced their prednisolone dosage until suspension due to clinical stability.

**Fig. 1:**
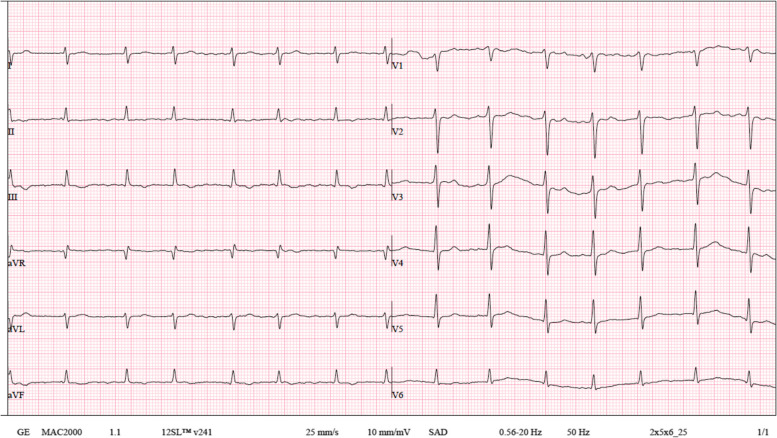
12-lead electrocardiogram. Atrial fibrillation rhythm, heart rate of 88 bpm, diffuse flattened T waves

**Fig. 2:**
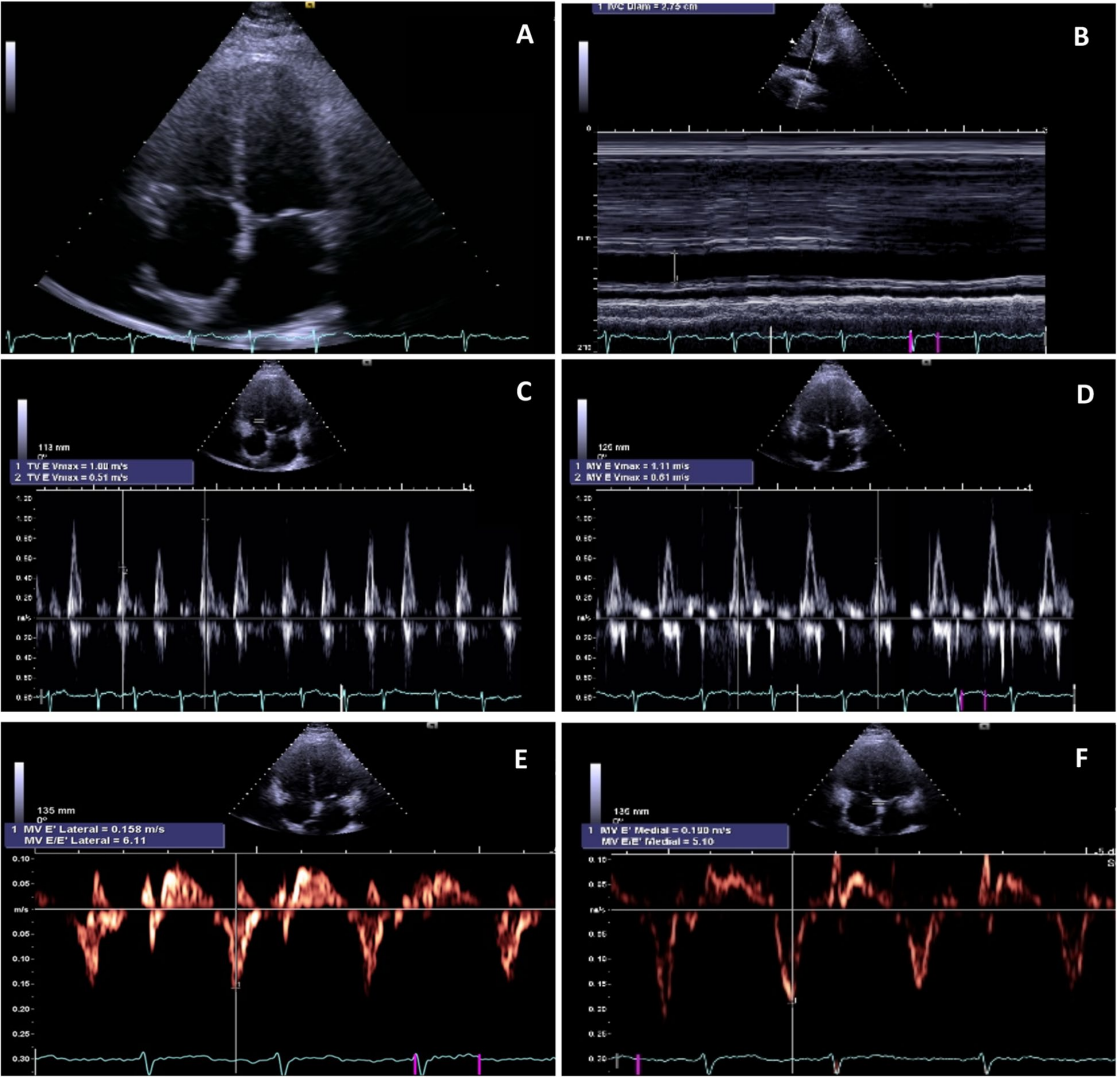
Transthoracic echocardiography performed during hospital stay. **A** Left and right atrial enlargement, pericardial thickness and effusion. **B** Inferior vena cava plethora. **C**, **D** Marked tricuspid and mitral valves inflow respiratory variations. **E**, **F** Pulsed wave tissue Doppler positioned at the lateral and septal mitral annulus

**Fig. 3:**
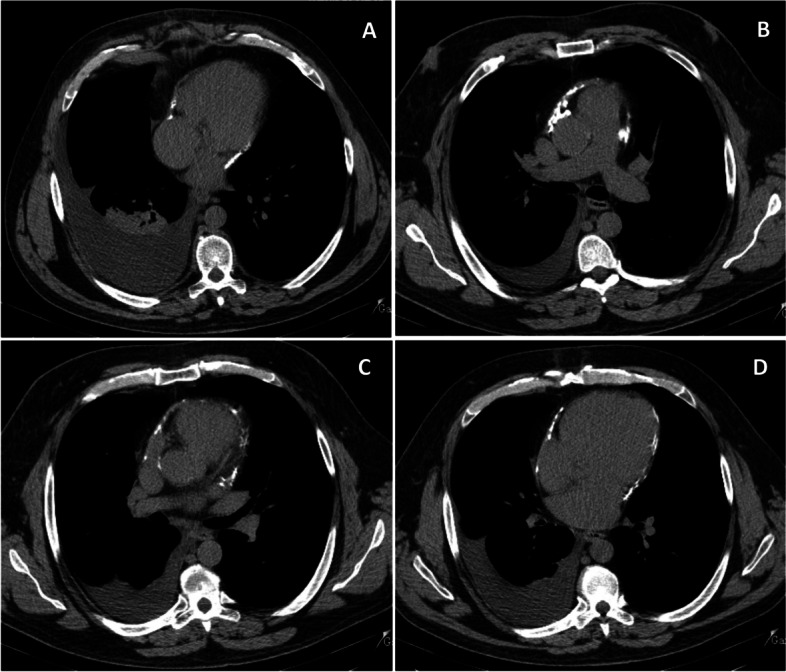
Chest computed tomography performed during hospital stay. **A** Right pleural effusion (6 cm) causing right lower lobe collapse. **B**, **C**, **D** Pericardial thickening with extensive calcified pericardial plaques

Four years after the first admission, the patient was readmitted with relapsed dyspnea, orthopnea and ascites. The physical exam was notable for jugular venous distension, lung base crackles, ascites and bilateral leg edema. Chest X-ray upon admission revealed a significant right pleural effusion with areas of passive atelectasis. The EKG showed atrial fibrillation rhythm with controlled ventricular rate of 86 bpm and T wave flattening in all precordial leads (Fig. 1). At this time a new TTE was performed showing left and right atrium enlargement (left atrial indexed volume = 43 mL/m^2^; right atrial indexed volume = 31 mL/m^2^), small ventricular cavities with mild left and right ventricular systolic dysfunction. It was also notable for an increased respiratory phasic septal shift as well as mitral and tricuspid flow variations. The additional presence of mitral *annulus reversus* and *annulus paradoxus*, inferior vena cava plethora and of a thickened echogenic pericardium with calcifications and a small pericardial effusion raised the clinical suspicion of constrictive pericarditis. (Fig. 2). A new thoracic CT scan was performed, showing a thickened pericardium with extensive calcified pericardial plaques (Fig. 3). No evidence of masses or lymphadenopathies suggestive of malignancy. Subsequent cardiac catheterization confirmed the diagnosis by demonstrating elevated and equal diastolic pressures in both ventricles, a “square root” sign during diastole and an elevated systolic area index. It also reveals isolated post-capillary pulmonary hypertension (Table 2).

**Table 2:** Hemodynamic parameters accessed during cardiac catheterization

Parameters	Measurement	Normal range
Systemic/ aortic pressure [s/d/m]	100 / 73 / 83 mmHg	140/90/105 mmHg
Left ventricular pressure [s/ed]	117 / 27 mmHg	100–140 / 3–12 mmHg
Right atrial pressure [mean]	22 mmHg	1—5 mmHg
Right ventricular pressure [s/ed]	48 / 26 mmHg	15–30 / 1–7 mmHg
PA pressure [s/d/mean]	47 / 30 / 37 mmHg	15–30 / 4–12 / 15 mmHg
PA wedge pressure [mean]	24 mmHg	4–12 mmHg
Cardiac index^a^	2.23 L/min/m^2^	2.5–4.2 L/min/m^2^
Systemic vascular resistance^a^	11.45 WU	10–20 WU
Pulmonary vascular resistance^a^	2.44 WU	< 2.5 WU

^a^Parameters determined using Fick method, *PA* Pulmonary artery

**Fig. 4:**
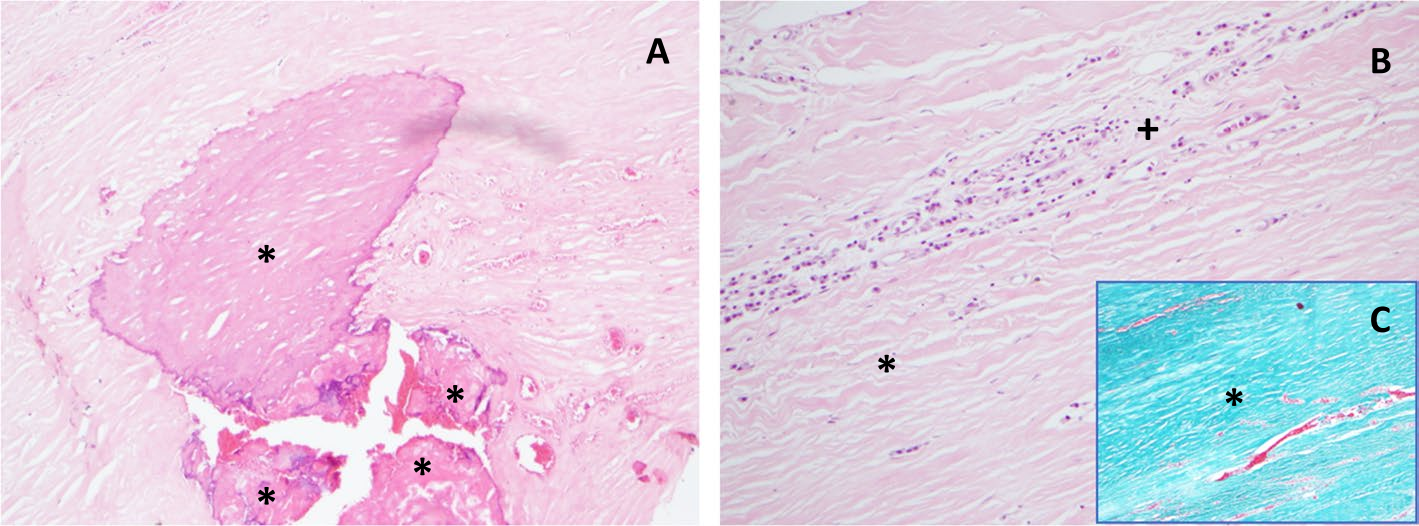
Explanted pericardial histopathologic tissue examination. **A** Hematoxylin and eosin (H&E) staining, 400 × magnification with central extensive areas of calcification. **B** H&E stain, 200 × magnification revealed hyaline fibrosis (*) and a scanty mononuclear inflammatory infiltrate ( +).**C** Masson´s trichrome staining, 200 × magnification, used for selective stain collagen fibers (*)

In face of this imaging features, the diagnosis of CP was confirmed. Due to the documented extensive calcifications it was assumed a chronic CP, so the patient was proposed and accepted for cardiac surgery with pericardiectomy. During the surgery, complex calcified adherences were evident around both ventricles and only a partial pericardiectomy was performed. The histologic tissue analysis revealed areas of hyaline fibrosis and calcification and a scanty mononuclear inflammatory infiltrate, suggestive of chronic pericarditis (Fig. 4). No evidence of granulomas, epithelial or lymphoid neoplastic tissue.

Since then, the patient referred clinical improvement with dyspnea and orthopnea relief, despite maintaining signs of peripheral congestion amenable with diuretic therapy. Imaging reevaluation with a TTE performed three months later still shows the same constrictive physiology signs that were present before surgical intervention.

## Discussion and conclusions

As it is known, pericarditis can be a common cardiac manifestation of certain autoimmune and autoinflammatory rheumatologic diseases, mainly SLE, RA and periodic fever syndromes, but is rarer in primary Sjögren’s syndrome [5, 6, 19]. The development of constrictive pericarditis can be a serious complication of an underlying autoimmune disease, and it may occur following untreated subclinical pericarditis in this context. Constrictive pericarditis is a rare and late complication of various clinical conditions characterized by chronic pericardial inflammation, which leads to thickening and calcification. While relatively uncommon, rheumatologic diseases can cause CP, typically resulting from a combination of immune-mediated mechanisms and chronic inflammatory states [1, 5, 6, 21]. However, the precise time interval between the initial insult and the development of constriction is variable and establishing a direct relationship between a particular etiology and the development of CP is challenging. In clinical practice, the etiological diagnosis is often presumptive [1, 3, 5].

Therefore, we have decided to summarize the available literature on pericardial involvement in various rheumatologic conditions and the cases where it has progressed to constrictive pericarditis (See Supplementary Table S1, Additional File 1). In SLE, acute pericarditis develops in 25% of patients and can occur at any stage of the disease. However, CP is a rare complication reported in less than 2% of SLE patients [5, 7, 8]. In RA, before the use of biologic agents, constrictive pericarditis was documented in 10–24% of all patients. However, currently RA is rarely associated with CP, and the cases described in the literature are often associated with disease flare-ups [5, 9, 10]. In SSc, CP is a rare finding and typically occurs in the late stages of the disease [5, 11, 12]. Acute pericarditis has been observed in medium- and small-vessel vasculitis, and to a lesser extent in large vessel vasculitis [5, 13–15]. Behçet’s disease has been reported to have cardiac involvement in up to 6% of cases, with pericardial involvement being the most common manifestation. Rare cases of constrictive pericarditis have also been reported [5, 23]. In other miscellaneous causes, including autoinflammatory syndromes, sarcoidosis and IgG4-related disease, acute pericarditis or clinically significant pericardial effusion are less commonly observed. Constrictive pericarditis can be an extremely rare complication in these conditions [5, 17, 18].

In our case, we excluded the main infectious, neoplastic and other autoimmune causes and made a diagnosis of pSS during the investigation. There were no clues suggesting other CTD such as SLE or RA, or a periodic fever pattern suggestive of an autoinflammatory condition, despite the presence of hepatosplenomegaly (which can also be a manifestation of some CTD other than pSS, mainly SLE and in Felty´s syndrome, although it may only be secondary to the congestive state). IgG4-related disease could represent an alternative etiology, but besides being rarer than pSS, typical involvement was not present, although IgG4-subtype testing was not available for full characterization. Although we cannot impute the causality of the CP to the pSS in our patient, we believe that both conditions can be related [5, 19, 21].

Pericardial involvement in pSS is uncommon compared to other rheumatologic diseases. It often remains asymptomatic and is typically detected through echocardiographic evaluation, with pericardial effusion being the main reported finding (prevalence around 8% according to some studies). Symptomatic cases include acute pericarditis or non-significant effusion [19–21].

We search medical literature and gathered case reports of symptomatic pericardial involvement in pSS. Our selection includes comprehensive case reports and case series detailing clinical presentation, diagnosis, treatment, and progression (See Supplementary Table S2, Additional file 1).

### This case raises important reflections in this context

Firstly, clinical suspicion and multimodality imaging play a crucial role in the diagnostic evaluation of suspected pericardial disease. The diagnosis of CP should be considered in patients with a clinical presentation predominantly related to right heart failure, including peripheral edema, ascites, pleural effusion and elevated jugular venous pressure (all symptoms reported in our case). Additional findings on physical examination, such as Kussmaul sign and pericardial knock, can reinforce clinical suspicion. The most relevant point in the diagnosis of constriction is considering it in the differential diagnosis when facing unexplained and recurrent ascites and requesting appropriate diagnostic tests [24–27]. Regarding complementary exams, transthoracic echocardiogram is recommended for initial evaluation. TTE provides several highly suggestive features that can assist in the diagnosis of constrictive pericarditis (as illustrated in Fig. 2). These important findings include respiratory-related ventricular septal bounce, respiratory variation in mitral and tricuspid inflow velocities, respiration-related hepatic vein flow reversals, and a distinct myocardial relaxation pattern represented by *annulus reversus*. The accuracy of echocardiography in diagnosing CP depends on which of these features are present, with reported sensitivity ranging from 64 to 87% and a specificity exceeding 90% [1, 3, 25]. A thoracic CT revealed pericardial thickening and determined the extent of pericardial calcification.

Albeit not routinely used for CP diagnosis, cardiac catheterization should be considered when previous non-invasive diagnostic methods have failed to confirm the diagnosis. This invasive procedure provides direct and simultaneous measurements of pressures in the cardiac chambers, pulmonary artery systolic pressure, and pulmonary capillary wedge pressure. In our case, cardiac catheterization was performed and confirmed our diagnosis [1, 3]. Cardiac magnetic resonance (CMR) although not useful to characterize pericardial calcifications, it could be helpful in assessing constrictive physiology and detecting active pericardial inflammation, which may respond to anti-inflammatory therapy. In our case, in the presence of extensive pericardial calcifications, a surgical approach was the only viable definitive therapy, making CMR unnecessary [3, 4, 27, 28].

Secondly, although not being the leading cause, CTD should be considered as a cause of constrictive pericarditis, particularly in young patients without other potential predisposition factors. In our patient, a definitive diagnosis of pSS was made with no other clinical manifestations than a mild sicca syndrome, successfully treated with no pharmacological measures. For the assumed pericardial involvement, systemic steroid therapy was ensued. Long-term immunosuppressive drugs can be considered in these patients to reduce relapses and steroid burden [21, 26, 27].

Finally, although constrictive pericarditis often poses a highly symptomatic burden to the patients, in our case, the sub-acute course of 6-years led to a late diagnosis of chronic CP. At this point, medical therapy can only provide supportive care to alleviate symptoms, and complete pericardiectomy is the only definitive therapeutic approach, although it may not always be feasible [4, 28, 29]. By the time the diagnosis was made, surgical treatment rendered suboptimal results as the patient, although reports clinical improvement, maintained signs of peripheral congestion and echocardiographic signs of constrictive physiology.

At the time of this report, and to the best of our knowledge, no other clinical case linking pSS to CP has been reported. Therefore, the authors hope to raise awareness about this challenging clinical association, which could lead to early diagnosis and improved medical management.

### Supplementary Information

**Additional file 1: Supplementary Table S2. **Published case reports of pericardial involvement in primary Sjögren’s syndrome. **Supplementary Table S2.** Published case reports of pericardial involvement in primary Sjögren’s syndrome.

## Data Availability

The datasets used and/or analyzed during the current study are available from the corresponding author on reasonable request.

## Abbreviations

CP: Constrictive pericarditis
CTD: Connective tissue diseases
SLE: Systemic lupus erythematosus
RA: Rheumatoid arthritis
SSc: Systemic sclerosis
pSS: Primary Sjögren´s syndrome
EKG: Electrocardiogram
TTE: Transthoracic echocardiography
CT: Computed tomography
CMR: Cardiac magnetic resonance
