# Supplementary material for: Chronic constrictive pericarditis: a rare cardiac involvement in primary Sjögren’s syndrome
Source: BMC Cardiovasc Disord. 2023 Sep 20;23:471. doi: 10.1186/s12872-023-03491-6 (PMC10512490; doi:10.1186/s12872-023-03491-6)
Supplement: Supplementary file 1 — Additional file 1: Supplementary Table S2. Published case reports of pericardial involvement in primary Sjögren’s syndrome. Supplementary Table S2. Published case reports of pericardial involvement in primary Sjögren’s syndrome. [file 12872_2023_3491_MOESM1_ESM.pdf]

Table S1: Available data regarding symptomatic pericardial involvement in rheumatologic disease.

Rheumatologic disease	Pericardial involvement (prevalence) (1)	Clinical manifestations	Constrictive pericarditis (2) (YES/NO)	Underlying mechanism	Treatment for pericardial involvement	Outcome(s)	Ref.
**Systemic lupus erythematosus (SLE)**	Acute pericarditis (25%)<br>Cardiac tamponade (<2%)	Symptomatic pericardial involvement occurs either at an early or late stage of the disease;<br>It is usually associated with other systemic manifestations;<br>Pericardial effusion is usually small and rarely cause hemodynamic compromise.	YES (<2%)	Immune complex-mediated reaction (complement C3 and IG deposits);<br>Genetic factors seem to be implicated.	1st line: NSAIDs or low to moderate dose of prednisone (0.3-0.5 mg/kg/day, p.o.)<br><br>2nd line: high dose of prednisone (1 mg/kg/day, p.o. or iv)<br><br>Recurrent pericarditis: chronic immunosuppression with MTX, AZP or MMF and IVIG	Pericardial involvement typically has a benign course and responds favorably to initial therapy.<br>Recurrent pericarditis is described.	[5,7,8,21]
**Rheumatoid arthritis (RA)**	Acute pericarditis (<10%)<br>Cardiac tamponade (rare)	Clinical pericarditis predominantly affects males and is associated with nodular forms of the disease and other extra-articular manifestations;<br><br>It often follows the onset of arthritis, but can also occur before the diagnosis of RA.	YES<br>Rare in the era of biologic agents;<br><br>Documented in 10-24% of patients in older studies.	Cytokines (TNFα, IL-1, and IL-6) and immune complexes deposition drive the immune pathway.	RA-associated pericarditis: prednisone (0.3-0.5 mg/kg/day, p.o.) tapered over 2-4 weeks;<br><br>Optimization of immunosuppressive drugs for RA (synthetic or biological DMARDs), mainly in flare-ups.	The impact of pericarditis on overall mortality is still uncertain.<br>Recurrent pericarditis is described.	[5,9,10,21]

**Systemic sclerosis (SSc)**	Pericardial effusion (~40%)<br>Acute pericarditis (~20%)<br>Cardiac tamponade (rare)	The presence of pericardial manifestations is commonly linked to the diffuse cutaneous subtype.	YES (rare)	Pericardial involvement may arise from microvascular dysfunction and autoimmunity.	Limited evidence supports specific therapies; caution is advised when using CCT (reported associations with unfavorable outcomes and recurrences); lacking information on the use of NSAIDs; IS drugs (MTX, CTX, MMF, AZP and IVIG) have been used with varying success rates.	Symptomatic cardiac involvement is associated with a poor prognosis, with a 5-year mortality rate of 75%.	[5,11,12,21]
**Vasculitis**							
**Takayasu arteritis (TA)**	Acute pericarditis (30%)	Pericardial involvement is uncommon as an initial manifestation of the disease. Recurrences are frequent, necessitating long-term IS therapy.	NO	Inflammation is mediated by cells and immune complexes.	1st line: (hd)CCT<br>2nd line: MTX; Biologic agents<br>Alternatives: CTX, AZP, MMF	TA is a chronic condition characterized by frequent relapses, with heart failure being the primary cause of death.	[5,16,21]
**EGPA**	Acute pericarditis (25%)<br>Pericardial effusion	Pericardial involvement can occur early or late in the course of the disease and typically accompanies systemic disease flares.	NO	Epicardial granulomas with eosinophilic infiltrates.<br><br>Cardiac damage is predominantly associated with ANCA negativity.	Most respond well to glucocorticoid therapy; its combination with other IS agents could be beneficial.	Cardiac involvement is one of the most significant predictors of adverse outcomes (given its frequently subclinical course).	[6,13,14,21]
**GPA**	Acute pericarditis, pericardial effusion (<10%)<br><br>Tamponade (rare)	Pericarditis is the most prevalent form of cardiac involvement and typically manifests in advanced stages of the disease.	YES (rare)	Autoinflammatory dysregulation (in the absence of active vasculitis or granulomatous infiltrate on the pericardium).	Most patients received a combination of CCT and CTX.	Cardiac involvement does not lead to worse outcomes, as the majority of patients respond favorably to standard therapy and do not experience clinical recurrences.	[5,14,21]

**Behçet´s disease**	Acute pericarditis (4-6%)<br>Pericardial effusion<br>Tamponade (very rare)	Chronic pericarditis and effusion are uncommon and can coexist with other cardiac complications.	YES (rare)	Inflammation mediated by cells and immune complexes.	1st line: CCT therapy<br>2nd line: CTX<br>Alternatives: Anti-TNFα therapy; Biologic agents	Depends on the severity of cardiac involvement; may require long-term IS therapy.	[5,23]
**Miscellaneous causes/AI syndromes**							
**FMF**	Acute pericarditis, pericardial effusion, cardiac tamponade (<4%)	Symptomatic pericarditis is relatively rare, and its clinical course varies.	YES (very rare)	Inflammasome activity triggered by the production of specific cytokines (functional mutation in the MEFV gene)	Colchicine is the mainstay treatment;<br><br>Alternative: CCT; Biologic agents (canakinumab, sc)	Depends on the severity of cardiac involvement.	[5,6]
**IgG4-RD**	Acute pericarditis, pericardial effusion (rare)	Pericardial involvement is seldom reported, and its presentation varies.	YES (very rare)	Pericardial infiltration by IgG4+ plasma cells (lymphoplasmacytic inflammation)	No consensus on optimal treatment;<br>1st line: CCT<br>2nd line: IS drugs (AZP, MMF, bortezomib)	Relapse is common after withdrawing CCT	[5,18]
**Sarcoidosis**	Acute pericarditis, pericardial effusion, tamponade (rare)	Symptomatic pericardial involvement is uncommon; typically associated with pericardial effusion and tamponade.	YES (very rare)	Immune complex-mediated reaction	1st line: (hd) CCT<br>2nd line: IS including MTX, AZP<br>3rd line: Biologic agents (anti-TNFα)	Cardiac sarcoidosis carries accounts is associated with higher morbidity and mortality rates.	[6,17]

AI: autoinflammatory; AZP: azathioprine; CCT: corticosteroids; CTX: cyclophosphamide; DMARDs: disease-modifying antirheumatic drugs; EGPA: Eosinophilic Granulomatosis with Polyangiitis; hd(CCT): high dose-CCT (1 mg/kg/d); IgG4-RD: IgG4-related diseases; IS: immunosuppressive; IVIG: Intravenous Immunoglobulin; GPA: Granulomatosis with Polyangiitis; MMF: Mycophenolate mofetil; MTX: methotrexate; NSAIDs: non-steroid anti-inflammatory drugs.

(1) Including symptomatic or clinically significant pericardial involvement. The prevalence is determined based on clinical and imaging findings reported in the literature, rather than relying on pathologic evidence from autopsies.

(2) "YES" if there are any case reports in the literature that specifically describe adverse progression to constrictive pericarditis.

Table S2: Published case reports of pericardial involvement in primary Sjögren's syndrome.

Author, Publication year, Reference	Sex, Age	Symptomatic pericardial involvement	Age at pSS diagnosis	Time from pSS diagnosis to pericardial involvement	Treatment for pSS	Treatment for pericardial involvement	Outcome
**Chen et al. 2009 [a]**	F, 57 yrs	Acute pericarditis	57 yrs	At the same time	Prednisone (high dose), CTX	Prednisone	In-hospital death (noncardiac complications)
**Mutsukura et al. 2007 [b]**	F, 35 yrs (pregnant)	Acute pericarditis	33 yrs	2 yrs later	Prednisone	Prednisone	Full recovery (no fetal complications)
**One et al. 2014 [c]**	F, 58 yrs	Acute pericarditis, pericardial effusion	58 yrs	At the same time	Prednisone (high dose)	Prednisone	Full recovery
**Rajani et al. 2013 [d]**	F, 50 yrs	Pericardial effusion	50 yrs	At the same time	Prednisone	Prednisone, pericardiocentesis	Improve
**Heper et al. 2017 [e]**	M, 70 yrs	Pericardial effusion	70 yrs	2 yrs before	Prednisone	Prednisone, pericardial window	Recurrent pericardial effusion attacks (before CCT initiation)
**Nayfeh et al. 2019 [f]**	M, 46 yrs	Acute pericarditis, pericardial effusion	46 yrs	6 months before	Prednisone, HCQ	NSAIDs, colchicine	Full recovery
**Medik Y et al. 2022 [g]**	F, 54 yrs	Cardiac tamponade	54 yrs	At the same time	MTX	Pericardiocentesis, pericardial window, CCT	Improve
**Abrams et al. 2018 [h]**	F, 27 yrs	Cardiac tamponade	27 yrs	At the same time	CCT; epoprostenol, PDE-5 inhibitor (PH)	Pericardial window	Improve
**Amaechi et al. 2021 [i]**	F, 50 yrs	Acute pericarditis, large pericardial effusion	50 yrs	At the same time	HCQ	NSAIDs, colchicine, pericardiocentesis	Full recovery
**Nair, P. 2021 [j]**	F, 69 yrs	Cardiac tamponade	69 yrs	3 months later	CCT, HCQ	Pericardiocentesis, CCT, colchicine	Initial recurrence; gradual recovery

*Our selection includes case reports and case series that provide comprehensive information on the clinical presentation, diagnosis, treatment, and clinical evolution of the subjects.

CCT: Corticotherapy; CTX: cyclophosphamide; HCQ: hydroxychloroquine; MTX: methotrexate; NSAIDs: non-steroid anti-inflammatory drugs; PH: pulmonary hypertension; PDE-5: phosphodiesterase 5; pSS: primary Sjögren’s syndrome; Yrs: years

(a) Chen, Hung-An et al. “Hemolytic uremic syndrome and pericarditis as early manifestations of primary Sjögren's syndrome.” *Clinical rheumatology* vol. 28 Suppl 1 (2009): S43-6. doi:10.1007/s10067-009-1121-3

(b) Mutsukura, Kazuo et al. “Successful treatment of a patient with primary Sjögren's syndrome complicated with pericarditis during pregnancy.” *Internal medicine (Tokyo, Japan)* vol. 46,14 (2007): 1143-7. doi:10.2169/internalmedicine.46.0062

(c) One, Masashi et al. “A case of primary Sjögren's syndrome with polyserositis.” *Eastern Journal of Medicine* 19 (2014) 54-57.

(d) Rajani, Ali et al. “An unexpected diagnosis in a dyspnoeic patient with primary Sjogren syndrome.” *BMJ Case Rep*. 2013, 2013:bcr2012007819. doi:10.1136/bcr-2012-007819

(e) Heper, Gulumser et al. “Sjögren’s Syndrome with Polyserositis, Gastrointestinal Findings and Ascending Aortic Aneurysm.” *Angiol* 2017, 5:2. DOI: 10.4172/2329-9495.1000193

(f) Nayfeh A. at al. “Sjogren’s Syndrome Presenting with Pleural and Pericardial Effusions.” *Am J Respir Crit Care Med* 2019;199:A3244. 10.1164/ajrccm-conference.2019.199.1_MeetingAbstracts.A3244

(g) Medik Y et al. “Cardiac tamponade-a rare complication of Sjogren’s syndrome.” *Am J Respir Crit Care Med*. 2022, 205:A1126. 10.1164/ajrccm-conference.2022.205.1_MeetingAbstracts.A1126

(h) Abrams, Hannah et al. “Pericardial effusion with cardiac tamponade and severe pulmonary hypertension as an initial presentation of primary Sjogren’s syndrome.” *Chest*. 2018, 154:4. 10.1016/j.chest.2018.08.822

(i) Amaechi, Eze et al. “Pericardial effusion as an atypical presentation of primary Sjögren syndrome.” *Crit Care Med*. 2021, 49:188. 10.1097/01.ccm.0000727476.12489.60

(j) Nair, Pooja, “Cardiac tamponade in Sjögren’s Syndrome.” *Journal of Hospital Medicine*. Abstract published at SHM Converge 2021. Abstract 588. https://shmabstracts.org/abstract/cardiac-tamponade-in-sjogrens-syndrome/. June 29th 2023.
